# Metastatic same-site squamous cell carcinoma arising during vismodegib therapy for basal cell carcinoma

**DOI:** 10.1016/j.jdcr.2022.07.032

**Published:** 2022-08-10

**Authors:** Raymond Zhao, Bo Wang, Lori Lowe, Andrzej Dlugosz, Christopher K. Bichakjian

**Affiliations:** a Department of Dermatology, University of Michigan Medical School, Ann Arbor, Michigan; b Department of Pathology, University of Michigan Medical School, Ann Arbor, Michigan; c Department of Cell and Developmental Biology, University of Michigan Medical School, Ann Arbor, Michigan; d Rogel Cancer Center, University of Michigan, Ann Arbor, Michigan

**Keywords:** basal cell carcinoma, same-site squamous cell carcinoma, vismodegib, BCC, basal cell carcinoma, SCC, squamous cell carcinoma

## Introduction

Basal cell carcinoma (BCC) is the most common human cancer worldwide. They typically arise due to ultraviolet radiation-induced mutations in *PTCH1* or *SMO* leading to uncontrolled activation of the Hedgehog signaling pathway, which underlies a majority of BCCs. A variety of treatment options may be considered for BCC based on tumor and patient characteristics. Vismodegib, a SMO receptor antagonist that downregulates the Hedgehog signaling pathway, is indicated for locally advanced BCC when surgery and radiotherapy are inappropriate and for metastatic BCC.^1^ However, with increased use of vismodegib for BCC, the development of concurrent squamous cell carcinoma (SCC) has been reported as a rare but potentially serious adverse effect.^2^, ^3^, ^4^ Herein, we present the first reported case of metastatic SCC developing during vismodegib treatment.

## Case report

**Fig 1:**
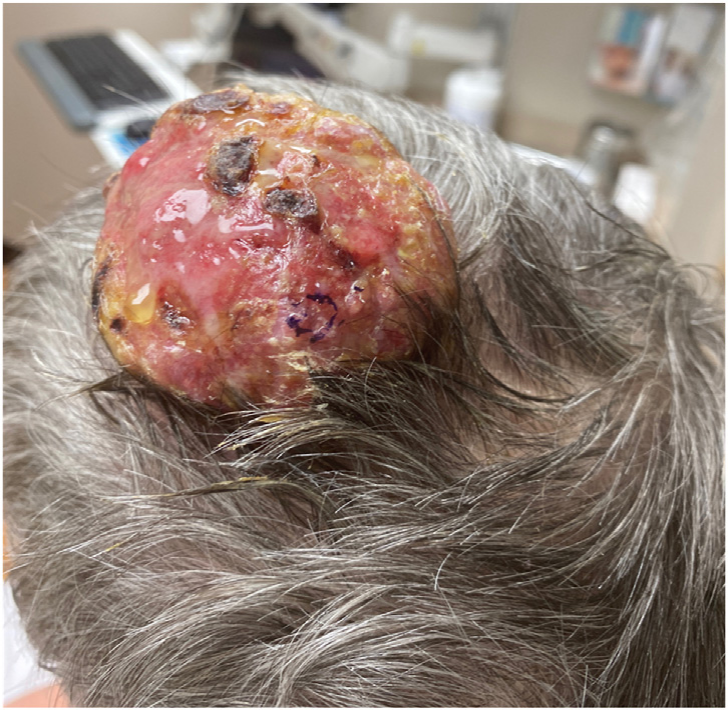
Squamous cell carcinoma. Large exophytic scalp mass after 9 months of vismodegib therapy.

A 56-year-old female on immunosuppressive therapy due to renal transplantation was referred to our clinic for a 7.0 × 6.5 × 2.5 cm firm, exophytic, erythematous, and ulcerated tumor on the left parietal scalp (Fig 1).

**Fig 2:**
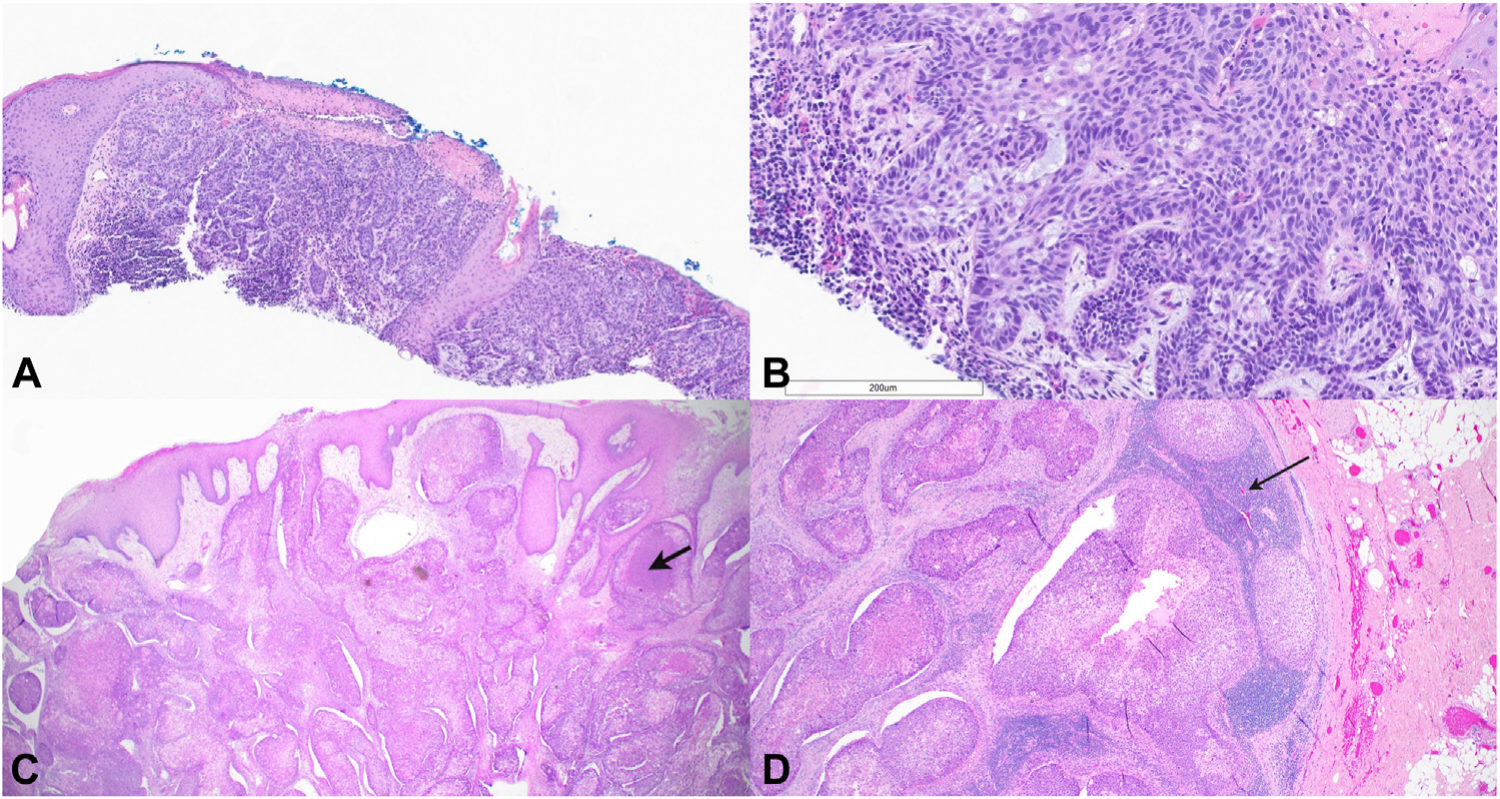
**A,** Shave biopsy of original BCC demonstrates an ulcerated epidermal surface with multiple basaloid tumor islands, variable peripheral palisading, and focal stromal retraction artifact (hematoxylin and eosin, 40×). **B,** Higher magnification of original BCC. Note the peripheral palisading and mucin within basaloid tumor islands (hematoxylin and eosin, 100×). **C,** Squamous cell carcinoma that developed in original BCC site. There are keratinizing tumor islands, variable clear cell features, and focal comedonecrosis (*arrow*) (hematoxylin and eosin; 100×). **D,** Metastatic SCC involving a left level 2B lymph node. Lymph node is replaced by keratinizing tumor islands of SCC. Only a small remnant of uninvolved lymph node tissue remains (*arrow*) (hematoxylin and eosin; 100×). *BCC*, Basal cell carcinoma; *SCC*, squamous cell carcinoma.

Nine months before presentation, she was seen at a local dermatologist’s office for a 3 cm friable plaque on her left vertex that had been slowly growing for the past 2 years. A skin biopsy was performed with a gross measurement of 0.7 × 0.4 cm. Histopathology demonstrated a BCC with circumscribed and infiltrative growth patterns (Fig 2, *A* and *B*). Given the size and location of the BCC, her dermatologist started her on neoadjuvant vismodegib 150 mg daily with a 2-weeks on, 2-weeks off schedule to reduce tumor size prior to surgery. Shortly thereafter, the patient noted enlargement of the tumor. However, due to a variety of reasons, she was not reevaluated by her dermatologist until 6 months after vismodegib initiation. When she eventually returned to her dermatologist, the lesion had significantly progressed in size and developed multiple erosions. This prompted a referral to our institution.

At the time the patient was evaluated in our clinic, she had completed 8 months of vismodegib. She endorsed rapid enlargement of the tumor and increased bleeding for the past 2 months. Importantly, both the referring dermatologist and the patient confirmed that the tumor was in the same location as the initial BCC. Based on the growth of the tumor while on therapy, vismodegib was promptly discontinued.

Due to this significant tumor appearance change since the initiation of systemic therapy, a repeat biopsy of the lesion was performed. This second biopsy demonstrated invasive, moderately differentiated SCC with clear cell features and areas of comedonecrosis (Fig 2, *C*). Positron emission tomography demonstrated uptake in the scalp mass, left parotid, and preauricular and upper cervical lymph nodes concerning for metastatic disease. Wide local excision of the primary scalp neoplasm was performed. Intraoperative frozen sections of a left neck lymph node were positive for metastatic SCC. Thus, a left superficial parotidectomy and left selective cervical lymph node dissection of levels II-IV were performed with 3 of 9 lymph nodes positive for metastatic SCC (Fig 2, *D*). Focal extranodal extension was present. The patient was additionally treated with adjuvant radiation therapy without immunotherapy.

## Discussion

Since Food and Drug Administration approval of vismodegib in 2012, there have been various reports describing the development of SCC during therapy with vismodegib for BCC.^2^, ^3^, ^4^, ^5^, ^6^, ^7^, ^8^, ^9^ A retrospective cohort study by Mohan et al^5^ found a significant hazard ratio of 8.12 for SCC development in patients receiving vismodegib compared with standard BCC therapy. However, after sampling a larger patient cohort (*n* = 1675 vs *n* = 180) and accounting for potential lead-time bias, a recent study by Bhutani et al^6^ was unable to identify an increased risk of SCC occurrence in patients treated with vismodegib. One distinct and rare phenomenon has emerged among the multiple reported cases of secondary SCC development while on vismodegib therapy. Similar to the patient we present here, a small subset of patients develops SCC(s) at the same site as their original BCC.^2^^,^^4^^,^^7^, ^8^, ^9^ To the best of our knowledge, this has only been observed in 5 previous case reports (Table I).

**Table I:** Reported cases of SCC that developed at the same site as the original BCC following vismodegib

Authors	Age/sex	BCC site	Vismodegib course	SCC presentation
Saintes et al^9^	76 M	Left face	3 mo	Progression of facial lesion despite therapy
	82 F	Nose	2 mo	Progression of a 0.3 cm lesion on nose
	49 F	Nose	3 mo	Development of 3 erythematous nodules around BCC
Zhu et al^4^	60s F	Right face	4 mo	Development of a 0.7 cm papule on BCC
	40s F	Scalp vertex	2.75 y	Progression of scalp tumor seen on MRI
Iarrobino et al^2^	61 M	Right shoulder with right axillary metastasis	Unspecified duration	Progression of axillary mass to a 3.5 cm fibrotic lesion
Poulalhon et al^7^	90 M	Nose	4 mo	Progression of BCC into a 5 cm granulated ulcerated lesion
Ransohoff et al^8^	62 F	Back with left axillary metastasis	13 mo	Development of a 3.5 cm mass in axilla

*BCC*, Basal cell carcinoma; *F*, female; *M*, male; *MRI*, magnetic resonance imaging; *SCC*, squamous cell carcinoma.

Clinical presentation of same-site SCCs varies in size and general morphology, ranging from small papules to large exophytic masses.^2^^,^^4^^,^^7^, ^8^, ^9^ In addition, the time course to development of SCC is variable. Clinical improvement or stability of the initial BCC prior to SCC development can be seen.^4^^,^^8^ However, metastatic disease has not been previously reported. Thus, the patient presented here demonstrates the most advanced presentation of secondary SCC reported to date, most notably with lymph node metastases. Possible explanations for the advanced stage may include the patient’s immunosuppressed status and the delay in presentation. Additionally, due to the small sampling size of the original lesion, there could have been a sampling error of the original BCC biopsy. In most reported cases, patients developed signs of secondary SCC within 4 months of initiation of therapy with vismodegib.^2^^,^^4^^,^^7^^,^^9^ Notably, even after an initial complete BCC tumor response to vismodegib, SCC can develop within a span of less than 4 months.^4^^,^^8^

The precise etiology of SCC development following inhibition of the Hedgehog pathway is not clear. It has been shown that same-site SCCs share many of the original tumor driver mutations as the original BCC, suggesting that these secondary malignancies develop from a BCC phenotypic switch rather than an independent *de novo* process.^8^ Kuonen et al^10^ propose that the loss of primary cilia in vismodegib-resistant BCCs may be the mechanistic link to SCC development. Relative cilia paucity in BCCs was associated with increased RAS/MAPK activity, a well-known oncogenic pathway in human SCCs.^10^

Despite its rare incidence and unclear pathogenesis, clinicians must be mindful of the potential adverse effect of same-site SCC development during vismodegib therapy for BCC. There should be a low threshold for repeat skin biopsy of treatment-resistant or growing lesions. Our case joins the growing body of evidence demonstrating SCC development during vismodegib therapy and highlights the importance of close clinical follow-up during BCC treatment.

## Conflicts of interest

None disclosed.
